# Regional differences in patient characteristics and outcomes during uninterrupted anticoagulation with dabigatran versus warfarin in catheter ablation of atrial fibrillation: the RE-CIRCUIT study

**DOI:** 10.1007/s10840-019-00518-x

**Published:** 2019-02-13

**Authors:** Stefan H. Hohnloser, Hugh Calkins, Stephan Willems, Atul Verma, Richard Schilling, Ken Okumura, Matias Nordaby, Eva Kleine, Branislav Biss, Edward P. Gerstenfeld, RE-CIRCUIT® investigators

**Affiliations:** 1 Department of Cardiology, J. W. Goethe University, Theodor-Stern-Kai 7, 60590 Frankfurt, Germany; 2 Electrophysiology Laboratory and Arrhythmia Service, Johns Hopkins Medical Institutions, Baltimore, MD USA; 3 Department of Cardiac Electrophysiology, University of Hamburg, Hamburg, Germany; 4 Department of Surgery, University of Toronto, Toronto, ON Canada; 5 Cardiac and Cardiology Research Department, St. Bartholomew’s Hospital, London, UK; 6 Division of Cardiology, Saiseikai Kumamoto Hospital Cardiovascular Center, Kumamoto, Japan; 7 TA Cardiology Medicine, Boehringer Ingelheim International GmbH, Ingelheim am Rhein, Germany; 8 Biostatistics + Data Sciences, Boehringer Ingelheim International GmbH, Ingelheim am Rhein, Germany; 9 Department of Clinical Operations, Boehringer Ingelheim RCV, Vienna, Austria; 10 Cardiac Electrophysiology and Arrhythmia Service, University of California, San Francisco, CA USA

**Keywords:** Ablation, Anticoagulation, Atrial fibrillation, Dabigatran, Regional difference, Warfarin

## Abstract

**Purpose:**

To describe regional differences in patient characteristics, ablation procedures, and bleeding events in the RE-CIRCUIT study. RE-CIRCUIT was a prospective, multicenter study that captured data from different regions, providing an opportunity to understand the practices followed in various regions. The incidence of major bleeding events (MBEs) was significantly lower with uninterrupted dabigatran versus uninterrupted warfarin.

**Methods:**

Patients were randomized to receive dabigatran 150 mg twice daily or warfarin. Ablation was performed with uninterrupted anticoagulation for 8 weeks after the procedure. Regions were Western Europe, Eastern Europe, North America, and Asia.

**Results:**

Of 704 patients screened across 104 sites, 635 underwent catheter ablation (dabigatran, 317; warfarin, 318). Patient characteristics were different across various regions. Patients from North America had the highest prevalence of atrial flutter (33%), coronary artery disease (29%), diabetes mellitus (18%), and previous myocardial infarction (9%). Hypertension was most prevalent in Eastern Europe (75%), as was congestive heart failure (40% vs 2% in Western Europe). Pulmonary vein isolation alone was the preferred technique used in most patients (86% in North America and 75–83% elsewhere) and radio frequency was the preferred energy source. The major outcome measure, incidence of MBEs during and up to 2 months after the procedure, was consistently lower with uninterrupted dabigatran versus warfarin, irrespective of regions and their procedural differences, and different ablation techniques utilized.

**Conclusions:**

This analysis shows that the benefits of dabigatran over a vitamin K antagonist in patients undergoing atrial fibrillation ablation are consistent across all geographic regions studied.

**Trial registration:**

NCT02348723 (https://clinicaltrials.gov/ct2/show/NCT02348723)

**Electronic supplementary material:**

The online version of this article (10.1007/s10840-019-00518-x) contains supplementary material, which is available to authorized users.

## Introduction

Atrial fibrillation (AF) is the most commonly sustained arrhythmia encountered in clinical practice. During the past decades, catheter ablation has evolved into an effective interventional treatment for AF [1–4]. However, the ablation procedure is associated with serious risks of periprocedural thromboembolic and bleeding complications [1]. Systematic periprocedural anticoagulation is, therefore, used to reduce the risk of stroke at the expense of an increased risk of bleeds. Currently, uninterrupted anticoagulation represents guideline-endorsed recommended anticoagulation strategy [1, 4, 5].

The Randomized Evaluation of dabigatran etexilate Compared to warfarIn in pulmonaRy vein ablation: assessment of a different peri-proCedUral antIcoagulation sTrategies study (RE-CIRCUIT® study) was an international multicenter study in which patients undergoing AF ablation were enrolled from many centers around the world. The study assessed the safety and efficacy of uninterrupted anticoagulation with dabigatran versus warfarin in patients undergoing catheter ablation of AF [6]. The primary study endpoint of International Society on Thrombosis and Haemostasis (ISTH) major bleeding events (MBEs) was observed significantly less commonly in the dabigatran treatment group than in the warfarin group (absolute risk difference − 5.3%; 95% confidence interval (CI) − 8.4 to − 2.2; *p* < 0.05) [6]. As RE-CIRCUIT was an international study including 104 centers in 11 countries, this database provides a unique opportunity to examine AF ablation techniques, patient characteristics, and outcomes globally.

## Methods

### Study design

RE-CIRCUIT was a prospective, randomized, open-label, blinded-adjudicated endpoint, multicenter, controlled study in patients scheduled for catheter ablation for paroxysmal or persistent AF (NCT02348723). The complete study design, methodology, and primary results were published previously [6, 7]. Briefly, eligible patients with paroxysmal or persistent AF with planned ablation of AF were randomly assigned to anticoagulation treatment with dabigatran etexilate 150 mg twice daily or international normalized ratio-adjusted warfarin. Ablation was performed with uninterrupted anticoagulation, continued for 8 weeks after the procedure [6]. Ablation procedures included pulmonary vein isolation (PVI), linear ablation, trigger ablation, and complex fractionated atrial electrograms. Energy sources, such as radio frequency, cryoballoon, and laser balloon, were used. Only PVI was pre-defined in the protocol, while additional ablation techniques and choice of energy source were at the discretion of the treating electrophysiologist. The study was performed in accordance with the provisions of the Declaration of Helsinki and the International Conference on Harmonisation Good Clinical Practice Guidelines [8, 9]. The study protocol and procedures were approved by relevant institutional review boards and ethics committees, and all patients provided written informed consent before entering the study.

### Patient population

Patients ≥ 18 years of age with paroxysmal or persistent non-valvular AF, with planned ablation of AF, and documented AF within 24 months before screening, as well as those who were eligible for treatment with dabigatran etexilate (150 mg twice daily), according to the local prescribing information, were eligible for the study. Full details of the RE-CIRCUIT inclusion/exclusion criteria have been published previously [6].

### Outcome measures

This subanalysis aims to evaluate the primary safety endpoint (the incidence of ISTH MBEs during and up to 2 months after the ablation procedure) across the different regions participating in RE-CIRCUIT. The secondary efficacy endpoint was the incidence of the composite of stroke, systemic embolism, or transient ischemic attack (TIA) during and up to 2 months after the ablation. The secondary safety endpoint was the incidence of minor bleeding events. Additionally, the incidence of bleedings requiring medical attention during and up to 2 months after the ablation procedure was analyzed in this post hoc analysis.

### Assessments and statistical analysis

For regional analysis, the geographic areas were classified into Western Europe, Eastern Europe, North America, and Asia. Western Europe included Belgium, France, Germany, Ireland, Italy, the Netherlands, Spain, and the UK. Eastern Europe included the Russian Federation. North America included Canada and the USA. Asia comprised Japan. As this was a post hoc analysis, data were analyzed using descriptive statistics. The outcome events were compared between regions and analyzed based on adjudicated data by a blinded adjudication committee from the start of the ablation procedure, considering a 2-month post-ablation period. Point estimates for the regional incidence of MBEs and their two-sided 95% CIs were determined, based on the exact method by Chan and Zhang [10].

## Results

### Study population

In the RE-CIRCUIT study, of 704 patients screened, 678 entered the study and 635 patients were administered at least one dose of the study drug and underwent the ablation procedure (dabigatran, 317 patients; warfarin, 318 patients) [6]. All 635 patients, consisting of 141 patients from North America, 329 from Western Europe, 57 from Eastern Europe, and 108 from Asia, were included in this analysis (Table 1).

**Table 1:** Patient baseline characteristics by region

	Western Europe	Eastern Europe	North America	Asia
Patients ablated, *n*	329	57	141	108
Age, mean, years (SD)	58.7 (10.0)	56.9 (9.1)	61.4 (10.8)	59.0 (11.2)
< 65 years, *n* (%)	232 (70.5)	45 (78.9)	80 (56.7)	69 (63.9)
65 to < 75 years, *n* (%)	84 (25.5)	12 (21.1)	45 (31.9)	35 (32.4)
≥ 75 years, *n* (%)	13 (4.0)	0 (0.0)	16 (11.3)	4 (3.7)
Male sex, *n* (%)	242 (73.6)	38 (66.7)	100 (70.9)	95 (88.0)
Body mass index, mean (SD), kg/m^2^	28.5 (5.1)	28.9 (4.2)	32.5 (7.9)	24.0 (3.3)
CHA_2_DS_2_-VASc score, mean (SD)	1.8 (1.2)	2.2 (1.1)	2.6 (1.8)	2.0 (1.1)
0, *n* (%)	27 (8.2)	3 (5.3)	11 (7.8)	2 (1.9)
1, *n* (%)	120 (36.5)	11 (19.3)	31 (22.0)	37 (34.3)
2, *n* (%)	94 (28.6)	22 (38.6)	39 (27.7)	42 (38.9)
> 2, *n* (%)	88 (26.7)	21 (36.8)	60 (42.6)	27 (25.0)
Medical history, *n* (%)				
Ejection fraction*, mean, ml (SD)	60.0 (7.4)	63.4 (7.2)	55.9 (11.5)	60.6 (9.7)
Left atrial size^†^, mean, mm (SD)	41.8 (6.3)	42.4 (4.4)	41.7 (6.9)	38.9 (5.4)
Atrial flutter	55 (16.7)	5 (8.8)	46 (32.6)	6 (5.6)
Congestive heart failure	8 (2.4)	23 (40.4)	29 (20.6)	5 (4.6)
Coronary artery disease	26 (7.9)	8 (14.0)	41 (29.1)	5 (4.6)
Diabetes mellitus	27 (8.2)	1 (1.8)	26 (18.4)	10 (9.3)
Hypertension	158 (48.0)	43 (75.4)	92 (65.2)	50 (46.3)
Previous MI^‡^	11 (3.3)	1 (1.8)	12 (8.5)	1 (0.9)
Stroke	7 (2.1)	3 (5.3)	4 (2.8)	5 (4.6)
Previous stroke, hemorrhagic	0 (0.0)	0 (0.0)	0 (0.0)	2 (1.9)
Previous stroke, ischemic	7 (2.1)	2 (3.5)	1 (0.7)	3 (2.8)
Previous stroke, uncertain	0 (0.0)	1 (1.8)	3 (2.1)	0 (0.0)
Systemic embolism	1 (0.3)	1 (1.8)	3 (2.1)	0 (0.0)
TIA	5 (1.5)	1 (1.8)	7 (5.0)	1 (0.9)
Atrial fibrillation, *n* (%)				
Paroxysmal	241 (73.3)	44 (77.2)	87 (61.7)	60 (55.6)
Persistent	80 (24.3)	11 (19.3)	46 (32.6)	30 (27.8)
Long-standing persistent	8 (2.4)	2 (3.5)	8 (5.7)	18 (16.7)

*MI* myocardial infarction, *SD* standard deviation, *TIA* transient ischemic attack

*Ejection fraction was missing in 21 patients

^†^Left atrial size was missing in 17 patients

^‡^Previous MI was missing in 5 patients

### Patient characteristics

In the RE-CIRCUIT study, baseline characteristics were well balanced between the treatment groups. However, there were distinct differences in patient characteristics across different geographic regions. Table 1 details baseline characteristics by region. The mean age of patients was 59.2 years, with patients from North America (mean age 61.4 years) tending to be older than those from other regions. The mean (standard deviation) stroke risk of patients, estimated by the CHA_2_DS_2_-VASc score, was higher in North America (2.6 (1.8)) than elsewhere (Eastern Europe 2.2 (1.1); Asia 2.0 (1.1); Western Europe 1.8 (1.2)). Patients with a higher prevalence of atrial flutter, coronary artery disease, diabetes mellitus, and previous myocardial infarction were more prevalent in North America (32.6%, 29.1%, 18.4%, and 8.5%, respectively), whereas hypertension (75.4%) and congestive heart failure (40.4%) were more prevalent in Eastern Europe. The mean baseline body mass index was highest in North America (32.5 kg/m^2^). Most patients had paroxysmal AF (68%). The percentage of patients with persistent or long-standing persistent AF was higher in Asia (44.4%) and North America (38.3%) compared with that in Western Europe (26.7%) and Eastern Europe (22.8%).

### Ablation procedure

PVI alone was the most widely used ablation technique overall (79.4% of all cases) and, in all regions, PVI was the preferred ablation technique (85.8% in North America and 75.0–82.5% elsewhere). Radio frequency was the most widely used energy source (62.9–75.0% of patients across all regions). The use of the cryoballoon technique was the highest in Western Europe (35.3%). Table 2 details procedural variables by region. Regional procedural differences by AF type are shown in Table 3. The average procedure duration was longer in North America than in other regions (mean duration: North America, 227.5 min; Western Europe, 175.9 min; Asia, 131.5 min; Eastern Europe, 117.8 min) (Table 3). PVI alone was the most widely used ablation technique, irrespective of AF type in all regions (paroxysmal: Western Europe, 84.6%; Eastern Europe, 81.8%; North America, 83.9%; Asia, 70.0%). Among other techniques, linear ablation (Western Europe, 10.3%; Eastern Europe, 7.0%; North America, 10.6%; Asia, 11.1%) and complex fractionated atrial electrogram (Western Europe, 11.2%; North America, 0.7%; Asia, 3.7%) were the major contributors (Supplementary Table 1).

**Table 2:** Procedural variables by region

Procedural variable	Western Europe	Eastern Europe	North America	Asia
Patients ablated, *n*	329	57	141	108
PVI only, *n* (%)	255 (77.5)	47 (82.5)	121 (85.8)	81 (75.0)
PVI plus, *n* (%)	72 (21.9)	5 (8.8)	18 (12.8)	26 (24.1)
Energy source RF, *n* (%)	207 (62.9)	37 (64.9)	104 (73.8)	81 (75.0)
Energy source cryoballoon, *n* (%)	116 (35.3)	15 (26.3)	27 (19.1)	10 (9.3)
Individual mean ACT during ablation, s				
Mean	341.3	304.3	330.5	341.4
Median	332.2	310.0	324.8	340.6
Q1	301.8	274.3	289.4	298.8
Q3	362.8	324.0	364.1	370.1

PVI plus = any other ablation in addition to PVI. Ablation type was non-PVI in 4 patients and data were missing in 6 patients. Energy source was laser balloon in one patient, other energy source in 28 patients, and data were missing in 9 patients. Mean ACT was missing in 15 patients

*ACT* activated clotting time, *PVI* pulmonary vein isolation, *Q* quartile, *RF* radio frequency

**Table 3:** Regional ablation procedures by AF type

	Western Europe		Eastern Europe		North America		Asia	
AF type and type of ablation	Dabigatran	Warfarin	Dabigatran	Warfarin	Dabigatran	Warfarin	Dabigatran	Warfarin
Mean length of ablation, min	175.9	175.9	117.8	117.8	227.5	227.5	131.5	131.5
Paroxysmal, *n*	118	123	22	22	41	46	32	28
Mean length of ablation, mean (SD), min	179.8 (114.8)	164.1 (103.3)	122.1 (44.5)	99.9 (40.8)	215.7 (112.3)	196.9 (96.9)	126.9 (55.2)	122.0 (29.5)
PVI only, *n* (%)	100 (84.7)	104 (84.6)	17 (77.3)	19 (86.4)	34 (82.9)	39 (84.8)	20 (62.5)	22 (78.6)
PVI plus, *n* (%)	17 (14.4)	19 (15.5)	2 (9.1)	1 (4.5)	5 (12.2)	7 (15.2)	11 (34.4)	6 (21.4)
Persistent, *n*	39	41	4	7	22	24	21	9
Mean length of ablation, mean (SD), min	173.5 (78.9)	201.4 (111.5)	120.0 (23.5)	136.6 (45.5)	239.2 (79.3)	267.9 (109.2)	139.4 (47.7)	125.9 (39.5)
PVI only, *n* (%)	27 (69.2)	22 (53.7)	4 (100.0)	6 (85.7)	19 (86.4)	22 (91.7)	16 (76.2)	9 (100.0)
PVI plus, *n* (%)	11 (28.2)	19 (46.3)	0	1 (14.3)	3 (13.6)	2 (8.3)	5 (23.8)	0
Longstanding persistent, *n*	6	2	1	1	2	6	9	9
Mean length of ablation, mean (SD), min	188.5 (103.6)	153.0 (29.7)	205.0 (−)	190.0 (−)	340.0 (18.4)	302.0 (30.9)	142.3 (44.7)	153.8 (42.6)
PVI only, *n* (%)	2 (33.3)	0 (0.0)	0 (0.0)	1 (100.0)	2 (100.0)	5 (83.3)	7 (77.8)	7 (77.8)
PVI plus, *n* (%)	4 (66.7)	2 (100.0)	1 (100.0)	0	0	1 (16.7)	2 (22.2)	2 (22.2)

PVI plus = any other ablation in addition to PVI. Ablation type was non-PVI in four patients and missing in six patients. Length of ablation was missing in three patients

*AF* atrial fibrillation, *PVI* pulmonary vein isolation, *SD* standard deviation

### Outcomes

**Fig. 1:**
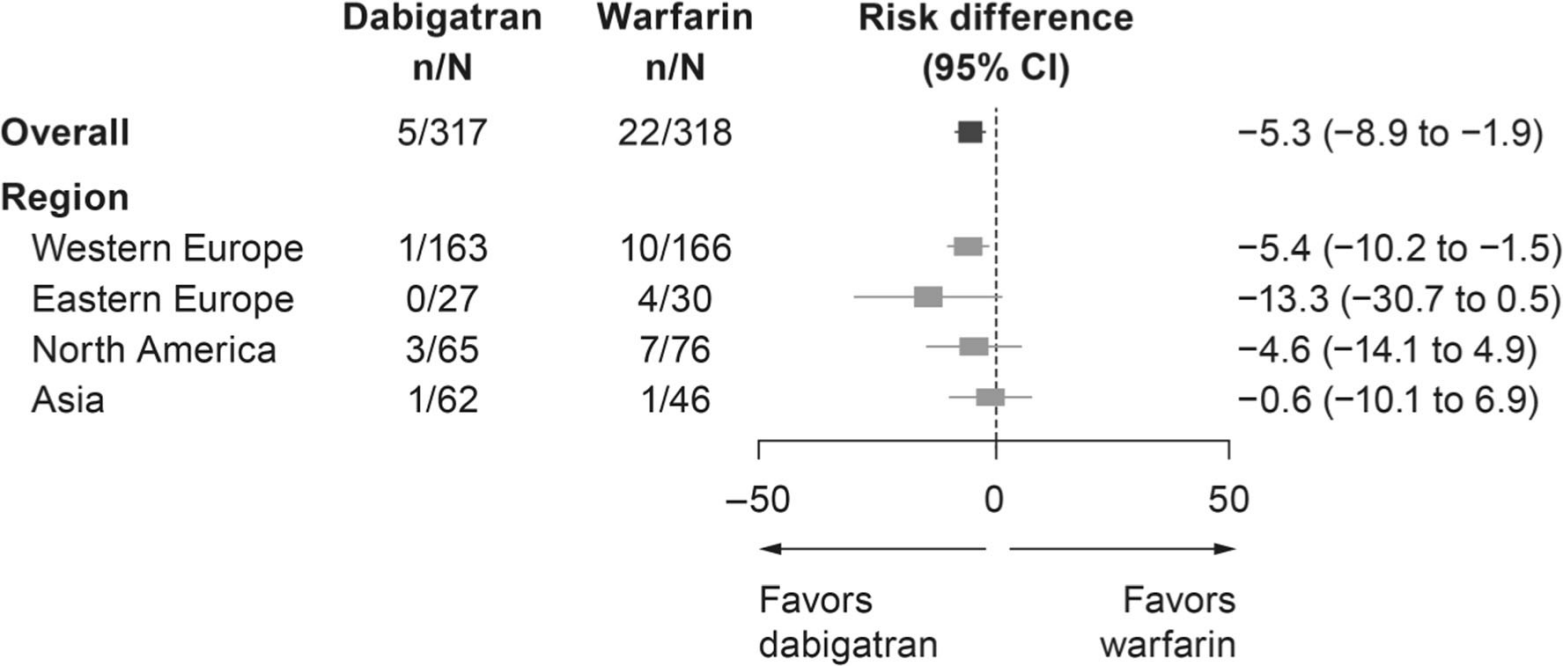
ISTH major bleeding events—regional distribution. *CI* confidence interval, *ISTH* International Society on Thrombosis and Haemostasis

In the overall study population, the incidence of the primary endpoint, ISTH MBEs, was lower in the dabigatran treatment group than that in the warfarin group (Fig. 1). This safety advantage with dabigatran was similarly observed for patients enrolled in North America, Western Europe, and Eastern Europe. In Asia, there were only two bleeding events (one patient in each treatment group). Bleeding events requiring medical attention were less frequently observed in those patients assigned to dabigatran than to warfarin who were enrolled in North America and Western and Eastern Europe, but not in Asia (Table 4).

**Table 4:** Numbers of patients with outcome events in the 2-month post-ablation period, and adverse events by region

	Western Europe		Eastern Europe		North America		Asia	
Patients	Dabigatran	Warfarin	Dabigatran	Warfarin	Dabigatran	Warfarin	Dabigatran	Warfarin
Patients ablated, *n*	163	166	27	30	65	76	62	46
ISTH major bleeds, *n* (%)	1 (0.6)	10 (6.0)	0 (0.0)	4 (13.3)	3 (4.6)	7 (9.2)	1 (1.6)	1 (2.2)
Minor bleeds, *n* (%)	34 (20.9)	35 (21.1)	1 (3.7)	0	11 (16.9)	10 (13.2)	13 (21.0)	9 (19.6)
Bleeds requiring medical attention, *n* (%)	14 (8.6)	24 (14.5)	1 (3.7)	4 (13.3)	6 (9.2)	11 (14.5)	12 (19.4)	6 (13.0)
Patients treated*, *n*	173	177	33	36	67	78	65	47
Patients with any AE, *n* (%)	129 (74.6)	124 (70.1)	14 (42.4)	18 (50.0)	43 (64.2)	62 (79.5)	39 (60.0)	38 (80.9)
Composite of stroke, systemic embolism, or TIA	0 (0.0)	0 (0.0)	0 (0.0)	0 (0.0)	0 (0.0)	1 (1.3%)	0 (0.0)	0 (0.0)

*41 treated patients did not start with the ablation procedure

*AE* adverse event, *ISTH* International Society on Thrombosis and Haemostasis, *TIA* transient ischemic attack

There were no composite events of stroke, systemic embolism, or TIA in the dabigatran group, while one TIA event was reported in the warfarin group. The incidence of minor bleeding events was also similar between treatment groups in all regions, with the lowest rates observed in Eastern Europe (1.8%). The regional distribution of any adverse event is shown in Table 4.

## Discussion

There are several new findings from the present post hoc analysis of the RE-CIRCUIT study. First, there are distinct differences in baseline characteristics of patients scheduled for AF ablation across various geographic regions, with more obesity and diabetes mellitus in North America, and more hypertension and congestive heart failure in Eastern Europe. Second, the vast majority of regions are performing PVI, predominantly using radio frequency energy. Third, the beneficial effects of dabigatran over warfarin in terms of bleeding events were observed consistently, irrespective of geographic region where patients were undergoing AF ablation.

The high prevalence of diabetes mellitus in North America may reflect the obesity epidemic in this region [11]. In Eastern Europe, a higher prevalence of hypertension and congestive heart failure may suggest less prevalent treatment for hypertension in this region, as well as reduced implementation of guideline recommendations [12, 13]. Uncontrolled high blood pressure increases the risk of stroke and bleeding events and may lead to a recurrence of AF [2, 3]. Patients from North America also had a higher mean CHA_2_DS_2_-VASc score than other regions, suggesting that North American patients may have a higher stroke risk compared with other regions. Addressing these regional differences in prevalence of obesity, hypertension, and congestive heart failure, therefore, remains important for reducing the burden of AF in the community.

Current guidelines recommend catheter ablation as a class 1 indication to improve AF symptom control only in patients with recurrent paroxysmal AF, despite anti-arrhythmic drug therapy. The guidelines recommend PVI as the cornerstone of AF ablation [3]. In the RE-CIRCUIT study, most ablation cases were paroxysmal AF (68%) [6], and PVI was the preferred ablation technique globally. Radio frequency was the most widely used energy source (67.6%) [6], followed by cryoballoon, most often reported in Western Europe. The widespread use of PVI suggests that guidelines for AF ablation have largely been followed throughout the world.

Ablation of persistent AF is known to have a lower success rate than that of paroxysmal AF and, so, some centers may employ adjunctive ablation techniques to improve the outcome [2]. In the present study, patients presenting with persistent or long-standing persistent AF were more common in Asia but less in Eastern Europe. Overall, even in patients with persistent AF, the dominant approach was PVI. This is reassuring given the results of the Substrate and Trigger Ablation for Reduction of Atrial Fibrillation (STAR-AF2) trial, which showed no benefit with linear or complex fractionated electrogram ablation in patients with persistent AF [14]. There was a tendency for centers in Asia and Western Europe to perform additional ablation outside PVI. Interestingly, despite a low usage of non-PVI ablation in North America (13.5%), procedures overall had a substantially longer duration in North America compared with Eastern Europe (227.5 vs 117.8 min). The reason for these differences is unclear, but it would be interesting to study whether there are regional differences in ablation techniques, and whether they translate into differences in procedural outcomes.

Despite these regional procedural differences, ablation was consistently associated with a lower bleeding risk in patients receiving dabigatran versus warfarin. The regional distribution of incidence of ISTH MBEs also favored the dabigatran treatment group, suggesting that the benefits of dabigatran over a vitamin K antagonist are mostly independent of the ablation technique, procedural differences, and patient characteristics [6]. This finding is also reflected by the lower incidence of bleeding events requiring medical attention, which was also consistently lower in the dabigatran group in all regions except Asia. Prior studies have suggested a higher bleeding risk from anticoagulation in Asian versus non-Asian patients [15].

### Limitations

The limitations of this analysis include the small sample size in some regions, and the inherent drawbacks associated with post hoc analyses. Our observations were made in a population suffering predominantly from paroxysmal AF. As such, the conclusions apply particularly for patients with this type of AF.

## Conclusions

In the RE-CIRCUIT study, uninterrupted therapy with dabigatran was associated with a significantly lower rate of MBEs than warfarin. The present post hoc analysis shows consistency of these observations, irrespective of the geographic regions and their procedural differences, and different ablation techniques utilized.

## Electronic supplementary material

Supplementary Table 1 (DOCX 20 kb)
