# Supplementary material for: Regional differences in patient characteristics and outcomes during uninterrupted anticoagulation with dabigatran versus warfarin in catheter ablation of atrial fibrillation: the RE-CIRCUIT study
Source: J Interv Card Electrophysiol. 2019 Feb 13;55(2):145–52. doi: 10.1007/s10840-019-00518-x (PMC6660508; doi:10.1007/s10840-019-00518-x)
Supplement: Supplementary file 1 — (DOCX 20 kb) [file 10840_2019_518_MOESM1_ESM.docx]

Supplementary Appendix

This appendix has been provided by the authors to give readers additional information about their work.

Supplement to: Hohnloser SH, Calkins H, Willems S, et al. Regional differences in patient characteristics and outcomes during uninterrupted anticoagulation with dabigatran versus warfarin in catheter ablation of atrial fibrillation: the RE-CIRCUIT study.

# Supplementary Table 1 Ablation type by region (ablation set)

|  | Western Europe  (*n*=329) | Eastern Europe  (*n=*57) | North America  (*n*=141) | Asia  (*n*=108) |
| --- | --- | --- | --- | --- |
| Ablation type, *n* (%) | | | | |
| PVI | 327 (99.4) | 52 (91.2) | 139 (98.6) | 107 (99.1) |
| Linear ablation | 34 (10.3) | 4 (7.0) | 15 (10.6) | 12 (11.1) |
| Trigger ablation | 2 (0.6) | 0 (0.0) | 1 (0.7) | 1 (0.9) |
| CFAE | 37 (11.2) | 0 (0.0) | 1 (0.7) | 4 (3.7) |
| Other | 25 (7.6) | 1 (1.8) | 3 (2.1) | 12 (11.1) |
| Atrial tachycardia | 1 (0.3) | 0 (0.0) | 0 (0.0) | 0 (0.0) |
| Cavotricuspid isthmus ablation | 15 (4.6) | 1 (1.8) | 2 (1.4) | 8 (7.4) |
| Left atrial roof | 6 (1.8) | 0 (0.0) | 1 (0.7) | 0 (0.0) |
| Mitral isthmus | 7 (2.1) | 0 (0.0) | 0 (0.0) | 0 (0.0) |
| Nodal tachycardia | 1 (0.3) | 0 (0.0) | 0 (0.0) | 0 (0.0) |
| SVC isolation | 1 (0.3) | 0 (0.0) | 0 (0.0) | 4 (3.7) |
| Missing | 0 (0.0) | 5 (8.8) | 1 (0.7) | 0 (0.0) |
| Other, right atrial procedure | 17 (5.2) | 1 (1.8) | 2 (1.4) | 12 (11.1) |
| Number of procedures, n (%) | | | | |
| 1 | 255 (77.5) | 47 (82.5) | 122 (86.5) | 82 (75.9) |
| 2 | 54 (16.4) | 5 (8.8) | 17 (12.1) | 24 (22.2) |
| 3 | 13 (4.0) | 0 (0.0) | 1 (0.7) | 2 (1.9) |
| 4 | 6 (1.8) | 0 (0.0) | 0 (0.0) | 0 (0.0) |
| 5 | 1 (0.3) | 0 (0.0) | 0 (0.0) | 0 (0.0) |
| Missing | 0 (0.0) | 5 (8.8) | 1 (0.7) | 0 (0.0) |

*CFAE* complex fractionated atrial electrogram, *PVI* pulmonary vein isolation, *SVC* superior vena cava.
